# Sources of persistent malaria transmission in a setting with effective malaria control in eastern Uganda: a longitudinal, observational cohort study

**DOI:** 10.1016/S1473-3099(21)00072-4

**Published:** 2021-11

**Authors:** Chiara Andolina, John C Rek, Jessica Briggs, Joseph Okoth, Alex Musiime, Jordache Ramjith, Noam Teyssier, Melissa Conrad, Joaniter I Nankabirwa, Kjerstin Lanke, Isabel Rodriguez-Barraquer, Lisette Meerstein-Kessel, Emmanuel Arinaitwe, Peter Olwoch, Philip J Rosenthal, Moses R Kamya, Grant Dorsey, Bryan Greenhouse, Chris Drakeley, Sarah G Staedke, Teun Bousema

**Affiliations:** a Department of Medical Microbiology, Radboud University Medical Centre, Nijmegen, Netherlands; b Department for Health Evidence, Radboud University Medical Centre, Nijmegen, Netherlands; c Infectious Diseases Research Collaboration, Kampala, Uganda; d Department of Medicine, University of California, San Francisco, CA, USA; e Department of Medicine, Makerere University College of Health Sciences, Kampala, Uganda; f Department of Infection Biology, London School of Hygiene and Tropical Medicine, London, UK; g Department of Clinical Research, London School of Hygiene and Tropical Medicine, London, UK

## Abstract

**Background:**

Symptomatic malaria cases reflect only a small proportion of all *Plasmodium* spp infections. Many infected individuals are asymptomatic, and persistent asymptomatic *Plasmodium falciparum* infections are common in endemic settings. We aimed to quantify the contribution of symptomatic and asymptomatic infections to *P falciparum* transmission in Tororo, Uganda.

**Methods:**

We did a longitudinal, observational cohort study in Tororo district, Uganda. We recruited participants of all ages from randomly selected households within this district. Participants were eligible if the selected household had no more than nine permanent residents and at least two members younger than 10 years, and the household was their primary residence, and they agreed to come to the study clinic for any fever episode and avoid antimalarial medications outside the study. Participants were followed-up by continuous passive surveillance for the incidence of symptomatic infections; routine assessments (ie, standardised clinical evaluation and blood samples) were done at baseline and at routine visits every 4 weeks for 2 years. *P falciparum* parasite density, gametocyte density, and genetic composition were determined molecularly using quantitative PCR (qPCR), quantitative reverse transcriptase PCR (qRT-PCR), and amplicon deep sequencing, respectively. Membrane feeding assays were also done to assess infectivity to mosquitoes. The contribution of different populations to the infectious reservoir was estimated for symptomatic infections, asymptomatic but microscopically detected infections, and asymptomatic but qPCR-detected infections; and for age groups younger than 5 years, 5–15 years, and 16 years or older.

**Findings:**

Between Oct 4, 2017, and Oct 31, 2019, 531 individuals were enrolled from 80 randomly selected households and were followed-up for 2 years. At baseline, *P falciparum* was detected in 28 (5·3%) of 531 participants by microscopy and an additional 64 (12·1%) by qPCR and declined thereafter. In 538 mosquito feeding experiments on 107 individuals, 446 (1·2%) of 37 404 mosquitoes became infected, with mosquito infection rates being strongly associated with gametocyte densities (β=2·11, 95% CI 1·62–2·67; p<0·0001). Considering both transmissibility of infections and their relative frequency, the estimated human infectious reservoir consisted primarily of asymptomatic microscopy-detected infections (83·8%), followed by asymptomatic submicroscopic infections (15·6%), and symptomatic infections (0·6%). Children aged 5–15 years accounted for more than half of the infectious reservoir (58·7%); individuals younger than 5 years (25·8%) and those 16 years or older (15·6%) contributed less. Samples from four children contribued to 279 (62·6%) of 446 infected mosquitoes after multiple mosquito-feeding assays.

**Interpretation:**

Individuals with asymptomatic infections were important drivers of malaria transmission. School-aged children contributed to more than half of all mosquito infections, with a small minority of asymptomatic children being highly infectious. Demographically targeted interventions, aimed at school-aged children, could further reduce transmission in areas under effective vector control.

**Funding:**

US National Institutes of Health, Bill & Melinda Gates Foundation, and the European Research Council.

## Introduction

Despite global efforts to control and eliminate malaria, progress has plateaued. In some high-transmission settings, malaria burden is increasing.^1^ Optimising vector control and treatment of symptomatic cases are central to malaria control and elimination efforts. However, symptomatic malaria cases reflect only a small proportion of all *Plasmodium* spp infections, and many infected individuals are asymptomatic. Asymptomatic infections are common in all malaria-endemic settings,^2^ although their prevalence is dependent on exposure to parasites, antimalarial immunity, use of preventive measures, and care-seeking behaviour.^3^ Asymptomatic infections with *Plasmodium falciparum* are particularly prevalent in school-aged children, who have a higher complexity of infection and more persistent parasitaemia than in younger children or adults.^4^, ^5^ In persistent infections, parasite densities might fluctuate over time, and are often below the threshold of detection by microscopy or conventional malaria rapid diagnostic tests.^2^, ^6^ These submicroscopic infections are disproportionately prevalent in areas of low endemicity and in settings that have reported a decline in malaria burden.^2^

**Research in context**

**Evidence before this study**

We searched PubMed for publications, without language restrictions, until Oct 1, 2020, using the search terms “plasmodium” AND “falciparum” AND “(“membrane feeding” OR “skin feeding” OR “direct membrane feeding” OR “mosquito feeding” OR “infectious reservoir”)” AND “(“longitudinal” OR “cohort”)”. We found one review of studies that assessed the infectious reservoir of malaria. This review identified ten studies done in 1955–2010 in different African and Asian settings. Nine of ten studies were done in areas of intense or moderate transmission intensity; none were longitudinal in study design. We also identified three additional recent cross-sectional studies, done between 2013 and 2016, that assessed the infectious reservoir in Burkina Faso and Kenya together, and in separate studies in Ethiopia and Cambodia. All three studies used mosquito membrane feeding assays and molecular assays to determine parasite and gametocyte densities. The study done in Burkina Faso and Kenya included only asymptomatic participants, whereas the two studies done in Ethiopia and Cambodia quantified infectivity to mosquitoes from both symptomatic and asymptomatic *Plasmodium falciparum* infected hosts. The study in Ethiopia observed that asymptomatic infections were responsible for 99·2% of all *P falciparum*-infected mosquitoes, whereas the study in Cambodia observed that only symptomatic infections resulted in *P falciparum* mosquito infections. The likelihood that an infection is interrupted by antimalarial treatment and the timing of this treatment relative to the appearance of infectious gametocytes are important factors in determining the relative contribution of symptomatic and asymptomatic infections. These factors are likely to be setting-dependent and are best examined in longitudinal studies. Only one study, done in Burkina Faso in 2007–08, did mosquito feeding experiments repeatedly on the same individual, with up to three observations per individual over 7 months. This study only included asymptomatic infections and did not examine the duration of infections but assessed gametocyte presence and infectivity in relation to seasonality.

**Added value of this study**

Our longitudinal study was done in an area of previously high transmission that is now under intense malaria control and as such is representative of other African settings where malaria burden has been successfully reduced. Our findings suggest that asymptomatic infections are responsible for the majority of mosquito infections with symptomatic infections having a negligible role in sustaining transmission in our setting. Excellent access to care in our cohort, demonstrated by a high proportion of gametocyte-free (short-duration) symptomatic infections might have contributed to these findings. In other settings, where symptomatic infections might be less likely to receive prompt treatment, these cases might develop into longer-lasting infections that produce transmissible gametocytes. Although microscopy-positive asymptomatic infections were responsible for most infected mosquitoes, parasite densities fluctuated considerably during infections such that these were frequently missed by research-level microscopy. We further identified school-aged children as an important and accessible reservoir that could be targeted by malaria control interventions.

**Implications of all the available evidence**

Recent studies have shown that asymptomatic low-density infections are common in areas of low endemicity and areas where control tools have reduced malaria burden. Our findings provide evidence that these asymptomatic infections, including low-density infections, are an important source of onward transmission to mosquitoes. Oscillations in the density of infections result in imperfect detectability by conventional diagnostics. Our findings show that although infectivity to mosquitoes is highest when asymptomatic infections are of sufficient density to be detected by microscopy, many infections that contribute to transmission are initially submicroscopic. This finding suggests that passive detection of symptomatic infections or community mass screening and treatment based on conventional diagnostics might be insufficient to reduce the infectious reservoir and prevent onward transmission in settings of declining transmission.

A considerable debate is ongoing about the characteristics of the human infectious reservoir. Understanding the relative contributions of symptomatic and asymptomatic infections to onward transmission of malaria parasites to mosquitoes is essential for guiding interventions to reduce and eliminate transmission.^7^, ^8^, ^9^ These contributions depend on parasite density, infection duration, care-seeking behaviour, and—importantly—gametocyte production. The likelihood that mosquitoes become infected is positively associated with gametocyte density.^10^ However, because of the long gametocyte maturation process, gametocytes are absent during the early stages of human infection. Gametocyte production might further vary between and during infections. Thus, an association between asexual parasite and gametocyte density might be absent in symptomatic infections and is weak in asymptomatic infections.^11^, ^12^, ^13^, ^14^

To better understand the contributions of different populations to the human infectious reservoir for malaria, we aimed to assess the density, genetic composition, and kinetics of naturally acquired *P falciparum* infections in an all-age cohort of residents in Tororo, Uganda. We also aimed to quantify gametocyte carriage and infectivity to mosquitoes, and compared them between age groups as well as participants with symptomatic and asymptomatic infections.

## Methods

### Study design and participants

We did a longitudinal, observational cohort study in Nagongera subcounty (Tororo district) in eastern Uganda. Tororo was previously a high-transmission area that is now under effective malaria control because of broad implementation of long-lasting insecticidal nets and indoor residual spraying of insecticides. Before 2013, malaria control in the Tororo district focused on malaria case management with artemether–lumefantrine, distribution of long-lasting insecticidal nets during antenatal visits, and promotion of intermittent preventive treatment during pregnancy. Universal distribution campaigns of long-lasting insecticidal nets were done in November, 2013, and May, 2017. Sustained indoor residual spraying of insecticides has been maintained since December, 2014, starting with rounds of bendiocarb every 6 months, followed by annual rounds of pirimiphos-methyl (Actellic; Syngenta, Basel, Switzerland) since June, 2016.^15^ Before implementation of indoor residual spraying of insecticides, the annual *P falciparum* entomological inoculation rate was 238 infectious bites per person per year, which was markedly reduced to 0·43 infectious bites per person per year 5 years later.^15^

In this context, from 2017 to 2019, we included participants of all ages into this study. Households were eligible if they had no more than nine permanent residents, at least two members younger than 10 years, no plans to move from Nagongera subcounty, and willingness to participate in screening for clinical and entomological surveillance. Households that met these criteria were selected; participants from these households were enrolled if their household was their primary residence and if they agreed to come to the study clinic for any episode of fever and avoid antimalarial medications outside the study.^15^

All eligible participants provided written informed consent. Ethical approval for the study was received from the Uganda National Council of Science and Technology (HS119ES), Makerere University School of Medicine, the University of California, and the London School of Hygiene & Tropical Medicine.

### Procedures

At baseline and at routine visits done every 4 weeks for 2 years, a standardised clinical evaluation was done and blood samples were collected. Participants were encouraged to attend a dedicated study clinic, which was open daily for all medical care. Study participants found to have a tympanic temperature of more than 38·0°C or a history of fever in the previous 24 h had a thick blood smear read urgently. If the thick blood smear was positive for malaria parasites by microscopy, samples were taken and individuals were treated with a 3-day course of artemether and lumefantrine (Coartem; Novartis, Kampala, Uganda). Asymptomatic infections were not treated unless symptoms occurred.

Blood samples drawn at the onset of each symptomatic malaria episode and at routine visits were assessed by quantitative PCR (qPCR) for *P falciparum* parasite density, targeting the multi-copy conserved var gene acidic terminal sequence, with a lower limit of detection of 0·05 parasite/μL using 200 μL whole blood samples.^16^ For all qPCR-positive samples, 100 μL of blood in RNA preservative (RNAprotect Cell Reagent; Qiagen, Hilden, Germany) was used for automatic extraction of nucleic acids to quantify male (PfMGET mRNA transcripts) and female (CCp4 mRNA) gametocytes by quantitative reverse transcriptase PCR (qRT-PCR) with a lower limit of detection of 0·1 gametocytes/μL.^17^ The primer and probe sequences are described in the appendix (pp 7–8). For the samples that were microscopically positive for gametocytes but qRT-PCR negative because of technical failure, microscopy gametocyte densities were used for analyses.

Mosquito membrane feeding assays were done on selected individuals who were diagnosed with symptomatic malaria before drug administration, or at the time of routine visits if an individual was qPCR positive at their previous visit. For each experiment, between 60–80 *Anopheles gambiae ss* mosquitoes were offered heparinised venous blood (Vacutainer Lithium Heparin Tube 4 mL; BD Biosciences, Plymouth, UK) via two glass feeders filled with 0·5 mL of blood each.^18^ 10 days after feeding, mosquitoes were dissected in 0·5% mercurochrome using a stereo microscope, and examined for the presence of oocysts on the mosquito midgut under an optic microscope by two microscopists (JO and AM); *P falciparum* DNA (18S) was detected in infected guts by nested PCR. To assess parasite diversity over the entire follow-up period, DNA from study participants and infected mosquito midguts was analysed by amplicon deep sequencing of the apical membrane antigen 1 (*AMA-1*) gene, as described previously.^18^ New infections were defined as the appearance of a new clone or group of clones at least 60 days after enrolment; to account for negative samples due to fluctuations in parasite density, we allowed three so-called skips in detection (absence in detection), and classified an infection as cleared only if it was not identified in four sequential samples from routine visits.^18^

Entomological surveillance was done throughout follow-up, with mosquito sampling every 2 weeks with Centers for Disease Control and Prevention light-traps in all rooms where cohort participants slept, and detection of *P falciparum* sporozoites in these mosquitoes with ELISA, as described elsewhere.^15^

### Statistical analysis

Poisson regression with generalised estimating equations to account for repeated measures was used to estimate incidence of symptomatic and asymptomatic infections and 95% CIs. Multiple generalised linear models were used to model measures of associations and present regression coefficients (β). Where appropriate, subject-specific random intercepts were added to account for correlations between observations from the same individuals. For response variables (gametocyte positivity and proportion of infected mosquitoes), we used generalised linear models assuming a binomial distribution with a logit for gametocyte positivity and a log link for the proportion of infected mosquitoes.

We modelled gametocyte prevalence using symptomatic status as a covariate including subject-specific random effects; the proportion of infected mosquitoes was modelled using the covariate gametocyte density.^10^ All continuous densities (ie, total parasite density and gametocyte density) were log transformed and a Gaussian distribution was used for the generalised linear model with an identity link, and subject-specific random effects were used. We modelled gametocyte density in two separate models, one using parasite density as a covariate and the other using age group as a covariate. Parasite density was modelled with the following covariates: age group, year, and their interaction.

The contribution of different populations to the infectious reservoir was estimated for symptomatic, asymptomatic but microscopically detected infections, and asymptomatic but qPCR-only detected infections;^9^ and for age groups younger than 5 years, 5–15 years, and 16 years or older. All visits were included in the calculations for contribution to the infectious reservoir by age group. For visits when mosquito feeding assays were done, the proportion of infected mosquitoes was known. For parasite-free visits, the proportion of infected mosquitoes was assumed to be zero. For parasite-positive visits during which no mosquito feeding assays were done but gametocyte density data were available, the proportion of infected mosquitoes was imputed using the relationship between gametocyte density and proportion of infected mosquitoes (appendix pp 4–6).

We did all the statistical analyses using R (version 3.1.12). Full details about the statistical analyses are shown in the appendix (pp 2–6).

### Role of the funding source

The funders of the study had no role in study design, data collection, data analysis, data interpretation, or writing of the report.

## Results

Between Oct 4 and Oct 31, 2017, 466 residents from 80 randomly selected households in Tororo were enrolled into the study cohort. An additional 65 residents were enrolled when they joined participating households during the follow-up period that ended on Oct 31, 2019. In total, from Oct 4, 2017, to Oct 31, 2019, 531 residents were enrolled and followed-up. 177 (33·3%) of 531 participants were younger than 5 years, 193 (36·3%) were aged 5–15 years, and 161 (30·3%) were 16 years or older. 62 (11·7%) of 531 participants were excluded from the study follow-up for the following reasons: moving out of the study area (n=41), absenteeism for more than 120 days (n=15), death (n=3), inability to comply with study protocol (n=2), or withdrawal of consent (n=1). Among the 469 participants actively in the study, 14 702 (99·6%) of 14 754 scheduled routine visits were successfully done.

**Figure 1:**
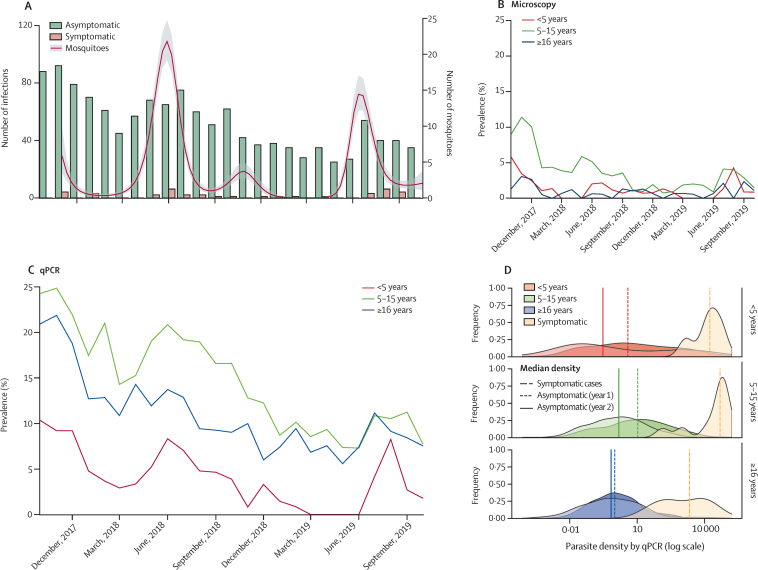
Symptomatic malaria episodes and asymptomatic *Plasmodium falciparum* infections (A) Numbers of asymptomatic qPCR-detected infections, symptomatic malaria infections, and mean number of mosquitoes caught per room per night. The line for mosquitoes represents a smoothed polynomial function, and the shaded area is the 95% CI. (B) Prevalence of *P falciparum* by microscopy for individuals at indicated ages. (C) Prevalence of *P falciparum* by qPCR. (D) Parasite density distributions by qPCR for different age groups. The vertical lines indicate median densities for asymptomatic infections by years 1 and 2, as well as for the symptomatic infections (ie, the 2 years combined) in the different age groups, as specified in the key. qPCR=quantitative PCR.

At baseline, parasites were detected by microscopy in 28 (5·3%) of 531 participants and by qPCR in an additional 64 (12·1%) participants. On average, two female *Anopheles* mosquitoes were collected per sleeping room per night, with peaks following seasonal rains (figure 1A). Of the 15 780 female *Anopheles* mosquitoes collected, nine (0·06%) were positive for *P falciparum* sporozoites. During the study follow-up, in which 12 728 qPCR assays were done, a decline in the number of asymptomatic infections detected per month was observed. Overall, 38 episodes of symptomatic malaria were diagnosed over 955 person-years (a clinical incidence of 0·040 episodes per person-year [95% CI 0·03–0·056]) and 110 new asymptomatic infections were detected (asymptomatic infection incidence of 0·12 episodes per person-year [95% CI 0·096–0·139]). Mean parasite prevalence over the study period in participants younger than 5 years was 1·2% by microscopy and 4·0% by qPCR, in those aged 5–15 years was 3·3% by microscopy and 14·0% by qPCR, and in participants 16 years or older was 0·8% by microscopy and 10·8% by qPCR (figure 1B, C). Geometric mean parasite density among symptomatic malaria infections was 10 825 parasites/μL (95% CI 5419–22 763), which was markedly higher than parasite densities among asymptomatic infections (2·67 parasites/μL, 95% CI 2·22–3·17; figure 1D). Among participants with asymptomatic infections, we observed lower parasite densities in the second year of follow-up than in the first year in individuals of all ages (mean difference in log densities between year 2 and year 1: <5 years −0·51 [95% CI −1·12 to 0·08; p=0·089]; 5–15 years −1·03 [–1·21 to −0·83; p<0·0001]; and ≥16 years −0·35 [–0·61 to −0·09; p=0·008]).

Overall, 538 mosquito feeding assays were done with blood samples from 107 individuals diagnosed with either symptomatic malaria (n=24 mosquito feeding assays) or asymptomatic infections (n=514). Of the feeds done on asymptomatic infections, 100 were done when individuals were parasite-negative, but with a positive qPCR result on the preceding visit (table). Parasite densities among participants selected for mosquito feeding assays were similar to those measured in the entire cohort (appendix p 10). At least one infected mosquito was observed in 39 (7·2%) of 538 feeding experiments, with 446 (1·2%) of 37 404 mosquitoes being infected. Of experiments that resulted in infected mosquitoes, 24 (61·5%) of 39 were from asymptomatic microscopy-positive individuals; this population was responsible for 389 (87·2%) of 446 infected mosquitoes, with far fewer infected mosquitoes arising from asymptomatic sub-microscopic infections (53 [11·9%] of 446) or symptomatic infections (four [0·9%] of 446; table).

**Table:** Mosquito feeding results

	**Number of observations**	**Number of feeds done**	**Number of infectious feeds**	**Number of mosquitoes dissected**	**Number of positive mosquitoes**
**All age groups**					
Total	14 701 (100%)	538	39 (7·2%)	37 404	446 (1·2%)
No parasites detected by qPCR	13 354 (90·8%)	100	0	7152	0
Sub-microscopic parasitaemia	1068 (7·3%)	313	14 (4·5%)	21 855	53 (0·2%)
Microscopically detected parasitaemia	241 (1·6%)	101	24 (23·8%)	6811	389 (5·7%)
Symptomatic malaria	38 (0·3%)	24	1 (4·2%)	1586	4 (0·3%)
**<5 years**					
Total	4111 (28·0%)	44	6 (13·6%)	2832	86 (3·0%)
No parasites detected by qPCR	3968 (27·0%)	18	0	1244	0
Sub-microscopic parasitaemia	94 (0·6%)	11	0	618	0
Microscopically detected parasitaemia	37 (0·3%)	8	6 (75·0%)	486	86 (17·7%)
Symptomatic malaria	12 (0·1%)	7	0	484	0
**5–15 years**					
Total	5910 (40·2%)	351	32 (9·1%)	24 466	350 (1·4%)
No parasites detected by qPCR	5149 (35·0%)	50	0	3692	0
Sub-microscopic parasitaemia	569 (3·9%)	206	14 (6·8%)	14 358	53 (0·4%)
Microscopically detected parasitaemia	172 (1·2%)	83	17 (20·5%)	5658	293 (5·2%)
Symptomatic malaria	20 (0·1%)	12	1 (8·3%)	758	4 (0·5%)
**≥16 years**					
Total	4680 (31·8%)	143	1 (0·7%)	10 106	10 (0·1%)
No parasites detected by qPCR	4237 (28·8%)	32	0	2216	0
Sub-microscopic parasitaemia	405 (2·8%)	96	0	6879	0
Microscopically detected parasitaemia	32 (0·2%)	10	1 (10·0%)	667	10 (1·5%)
Symptomatic malaria	6 (<0·1%)	5	0	344	0

Data are n or n (%). A median of 69 (IQR 62–81) mosquitoes were examined for infection status per feed. The proportion of all observations reflects the occurrence of stated infection status and age group among all observations. qPCR=quantitative PCR.

**Figure 2:**
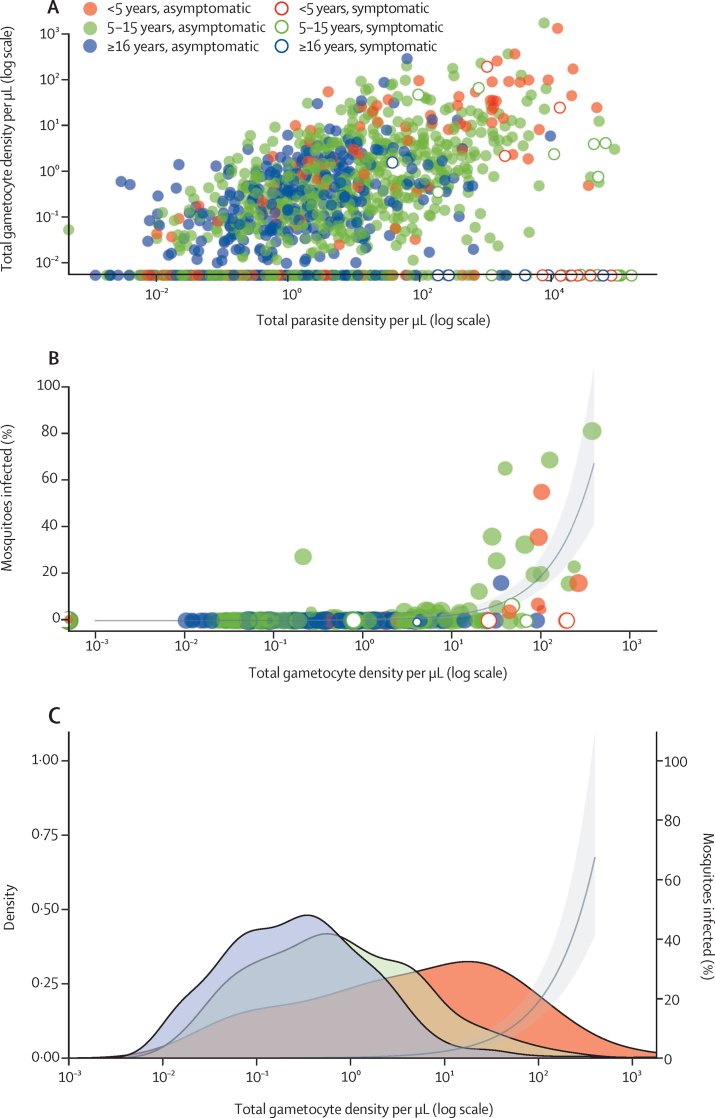
Gametocyte prevalence and density in relation to infectiousness to mosquitoes (A) Relationship between total parasite density and total gametocyte density. Each symbol is a parasite-positive episode. (B) Percentage of infected mosquitoes in relation to gametocyte density. The size of the symbols reflects the number of mosquitoes dissected. The line represents the best-fitted association and the shaded area is the 95% CI. (C) Gametocyte density by qRT-PCR among individuals who were gametocyte positive in different age groups. The line represents the best-fitted association and the shaded area is the 95% CI. qRT-PCR=quantitative reverse transcriptase PCR.

Gametocyte prevalence by qRT-PCR was significantly higher in asymptomatic infections than in symptomatic infections (884 [67·6%] of 1308 *vs* 11 [28·9%] of 38; odds ratio [OR] 3·2, 95% CI 1·1–9·3; p=0·033). 18 (46·2%) of 39 infectious feeds and 273 (61·2%) of 446 infected mosquitoes were from individuals with microscopically detectable gametocytes at the time of mosquito feeding. Among samples that were both positive for parasites and gametocytes (n=895), gametocyte density was positively associated with concurrent total parasite density in asymptomatic infections (β=0·30, 95% CI 0·26–0·34; p<0·0001), but not in symptomatic infections (β=–0·003, −0·39 to 0·39; p=0·903; figure 2A). Gametocyte density was strongly predictive of the proportion of mosquitoes that became infected when feeding on a blood meal (β=2·11, 95% CI 1·62–2·67; p<0·0001; figure 2B). Although no evidence for an interaction effect was reported between age and gametocyte density on the proportion of mosquitoes infected (interaction β=0·09, 95% CI −0·02 to 0·20; p=0·115), gametocyte densities among gametocyte carriers differed between age groups (figure 2C; appendix p 11). The median gametocyte density among gametocyte carriers overall was 4·89 (IQR 0·49–28·35) for children younger than 5 years, 0·66 (0·15–3·12) for those aged 5–15 years (p<0·0001), and 0·28 (0·07–0·89) for children 16 years or older (p<0·0001).

**Figure 3:**
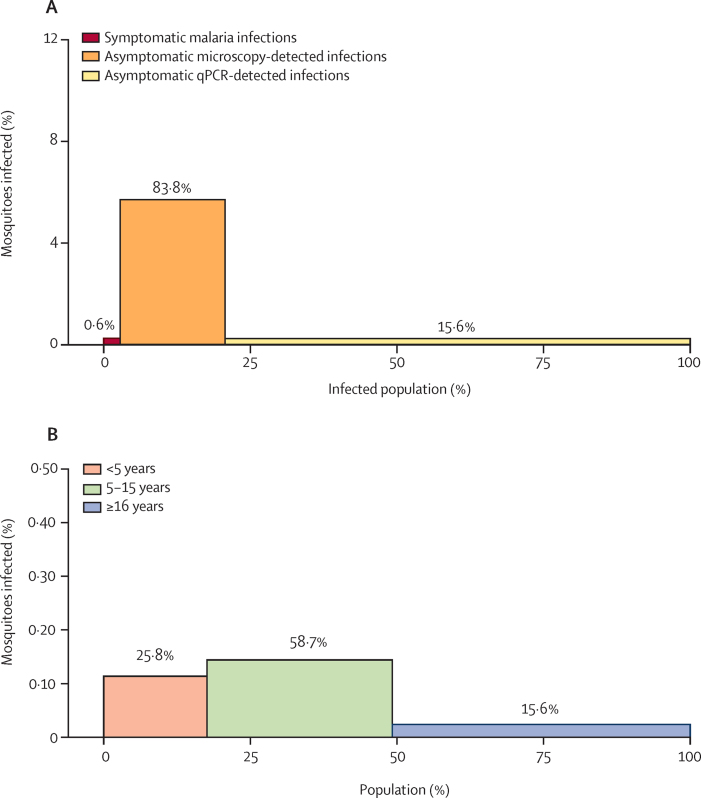
Contribution of different populations to the human infectious reservoir for malaria The bar heights indicate the proportion of mosquitoes that became infected when feeding on this population. The bar widths indicate the proportion of the infected population. (A) The contribution of different infection types to the infectious reservoir in the infected population. (B) The contribution of different age groups to the human infectious reservoir at a population level.

Considering both transmissibility of infections and their relative frequency in our study cohort, asymptomatic microscopy-detected infections were estimated to be responsible for 83·8% of the infectious reservoir, with asymptomatic qPCR-detected infections being responsible for 15·6% and symptomatic infections being responsible for 0·6% (figure 3A). We estimated the contribution of different age groups to the infectious reservoir based on mosquito feeding results and, when no feeding was done, parasite and gametocyte density measures to model infectiousness to mosquitoes. We estimated that children aged 5–15 years contributed to 58·7% of the infectious reservoir, followed by children younger than 5 years (25·8%), and those 16 years or older (15·6%; figure 3B; appendix pp 4–6).

**Figure 4:**
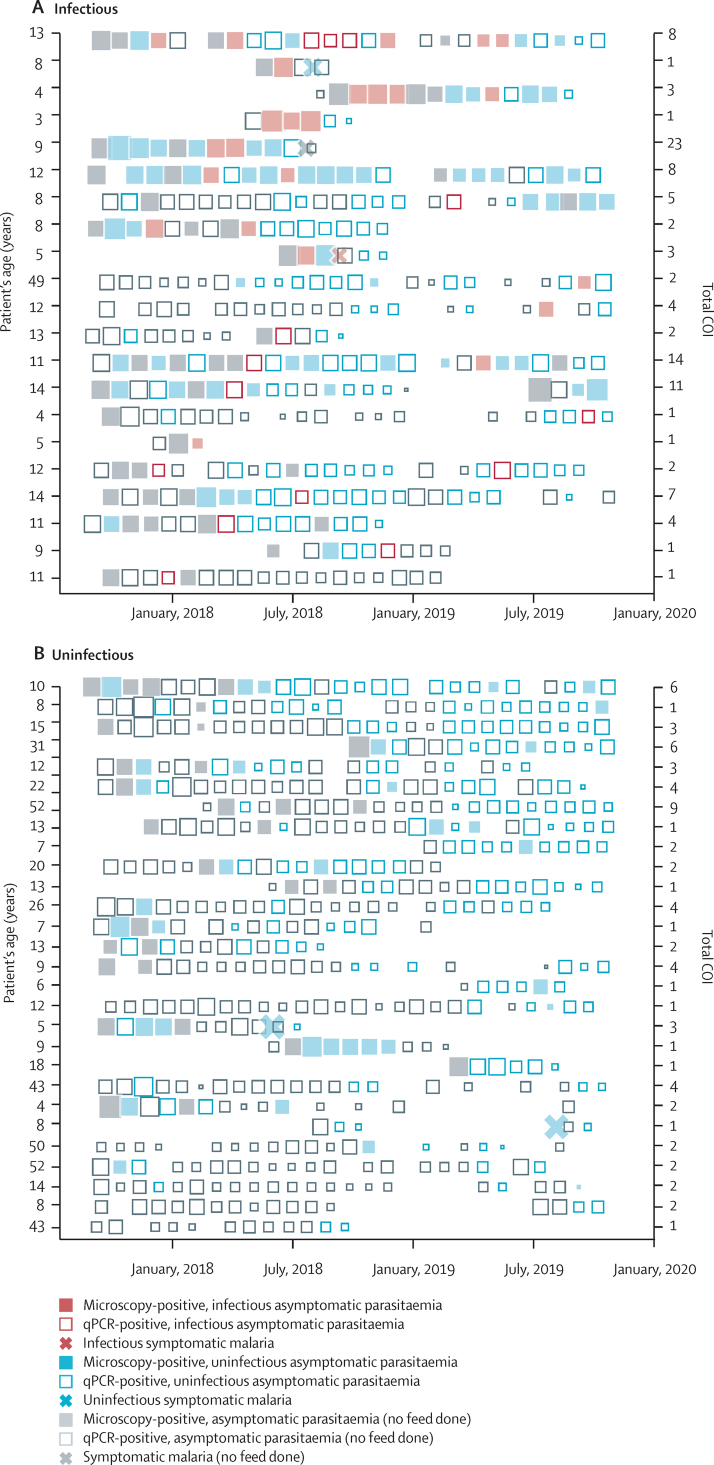
Longitudinal infectivity to mosquitoes Each row represents a cohort participant followed-up for 24 months. Each square indicates a visit when parasites were detected, with the size of the square reflecting qPCR parasite density. (A) Contains all individuals who were infectious on at least one occasion, ranked from top to bottom based on the total number of mosquitoes they infected. (B) Contains a selection of individuals, chosen from 86 individuals, who were never infectious to mosquitoes but had repeated feeding assays. qPCR=quantitative PCR. COI=complexity of infection.

Overall, 75 individuals participated in multiple mosquito feeding assays (median 6, IQR 2·5–9·0). Although 14 (35·9%) of 39 infectious feeds were done on samples from individuals with parasites below the concentration detected by microscopy, all infectious individuals were microscopy-positive for asexual parasites or gametocytes at least once during follow-up (figure 4A). Four (0·8%) of 531 individuals contributed to 279 (62·6%) of 446 infected mosquitoes. These four highly infectious individuals were aged 3 years, 4 years, 8 years, and 13 years; two of whom (one aged 4 years and one aged 13 years) had clonally complex infections (up to 23 clones detected during follow-up). Three of these children (aged 3 years, 4 years, and 8 years) acquired infections during follow-up that were transmissible in the absence of symptoms. 28 individuals with parasitaemia never infected mosquitoes despite five mosquito feeds or more; of whom, 22 (78·6%) had infections documented by qPCR for more than 12 months (figure 4B).

**Figure 5:**
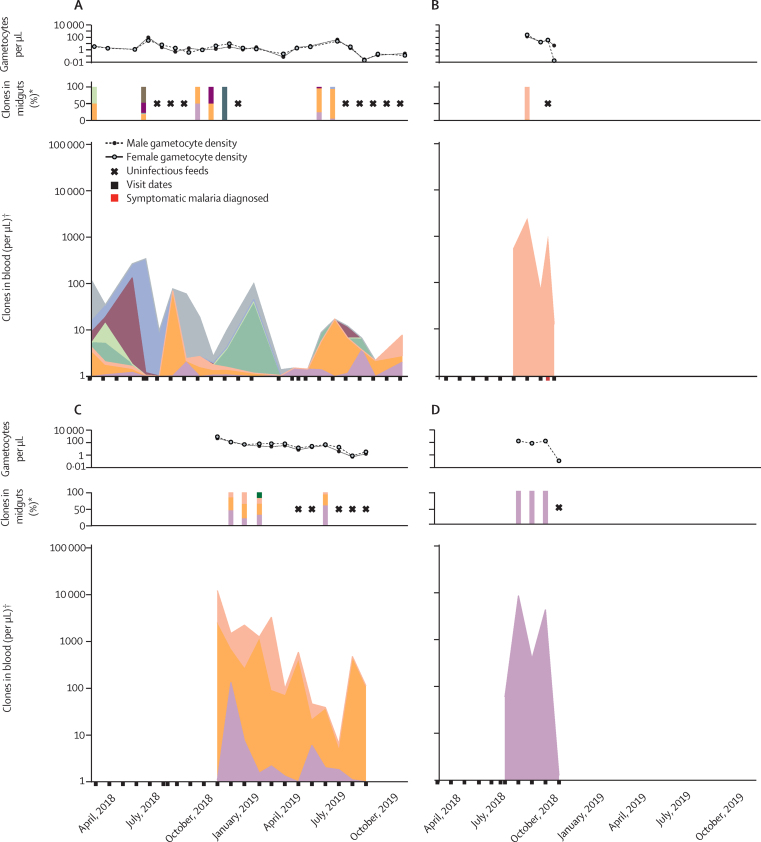
Gametocyte density and parasite clones recovered from blood and mosquito midguts in the four most infectious individuals Each individual colour indicates the contribution of a unique *Plasmodium falciparum* clone. Male and female gametocyte densities and the number of clones detected in blood and infected mosquitoes are shown for samples from the four individuals who infected the most mosquitoes in all mosquito membrane feeding experiments combined. *The bars indicate infectious feeds done during the visit dates. †Total parasite density and clonal composition of blood samples.

*P falciparum* DNA was detectable by nested PCR in 414 (92·8%) of 446 infected mosquito midguts; amplicon deep sequencing was successful for 322 (72·2%) of the 446 samples, providing genotyping data from at least one mosquito for 33 (84·6%) of 39 successful feeds. Identification of genotypes over time, in relation to transmission to mosquitoes, is presented for the four most infectious individuals in the cohort (figure 5); graphs for other participants are provided in the appendix (pp 12–17)*.* Two genetically complex infections repeatedly transmitted multiple clones to mosquitoes (figure 5A, C); two single-clone infections resulted in short periods of high infectivity to mosquitoes (figure 5B, D). Overall, 90·5% of parasite clones that were transmitted to mosquitoes were detected in the blood of the participant on the day of mosquito feeding; 6·6% were only detected in the blood on previous visits, and 2·9% were never detected in the blood of that participant. A strong positive association was observed between the abundance of a clone in peripheral blood and the abundance recovered in infected mosquitoes (β=0·43; p<0·0001; appendix p 18).

## Discussion

To effectively eliminate malaria, the human infectious reservoir must be targeted. However, very few studies have quantified this reservoir, and those that did were largely cross-sectional in study design, ignoring the duration of asymptomatic infections and fluctuations in parasite densities over time.^2^, ^6^, ^19^ In this longitudinal study, we identified the *P falciparum* infection status, and measured the gametocyte carriage and transmissibility over 2 years in an area of Uganda where malaria transmission was previously high, but is now low because of highly effective malaria control.

Asymptomatic parasite carriers comprised more than 99% of the human infectious reservoir for malaria in our setting. Within this asymptomatic population, parasite density was positively associated with gametocyte density,^20^ and those individuals with microscopically detectable infections contributed to 83·8% of the infectious reservoir. Symptomatic malaria infections were uncommon (0·040 episodes per person-year) and comprised only 0·6% of the human infectious reservoir. This very small contribution of symptomatic malaria infections to transmission is consistent with recent estimates for *P falciparum* transmission in Ethiopia,^9^ but markedly different from findings in Cambodia and Thailand, where symptomatic malaria cases with high gametocyte densities were suggested to be more important than asymptomatic malaria infections for sustaining transmission.^7^, ^8^ These contrasting findings might, in part, be attributable to treatment-seeking behaviour. In our cohort, symptomatic malaria episodes were less likely, compared with asymptomatic infections, to be gametocytaemic on presentation. This result suggests that most symptomatic malaria cases presented early, before the 9–12 day maturation of gametocytes was completed.^21^, ^22^ Gametocyte commitment might also be lower early in symptomatic infections (when parasites invest more in asexual multiplication) compared with asymptomatic infections, and gametocyte infectivity might be reduced because of gametocyte-inactivating activity associated with inflammation. In asymptomatic infections, parasite density, which is strongly associated with immunity of the infected individual,^5^, ^23^, ^24^ is likely to be crucial in determining transmissibility. Parasite densities that were above the threshold of detection by microscopy were relatively common in children within our cohort, who frequently had concurrent gametocyte densities high enough to result in mosquito infection. In some settings of very low transmission intensity, the majority of asymptomatic infections persist with ultra-low densities that are probably incompatible with transmission to mosquitoes.^12^

Although our study setting was historically characterised by intense transmission,^15^, ^25^ many of our cohort participants were born after indoor residual spraying of insecticides was implemented in the area and malaria exposure dropped to very low levels.^15^ Some of these young children acquired incident infections without having symptoms, supporting earlier indications that children exposed to low transmission can acquire immunity very efficiently.^23^ Several of these incident asymptomatic infections were highly important for transmission to mosquitoes. We hypothesise that if transmission remains low, ultimately a larger fraction of incident infections will result in symptoms and might be detectable by passive surveillance.

We observed marked differences in parasite carriage, parasite density, and transmission potential between age groups. Parasite prevalence by microscopy and qPCR as well as parasite densities were highest among children aged 5–15 years (appendix p 11).^4^, ^5^, ^26^ When combining the age distribution in the study district with parasitological and transmission data, individuals younger than 5 years were estimated to comprise 25·8%, children aged 5–15 years comprised 58·7%, and those 16 years or older comprised 15·6% of the human infectious reservoir. This finding is in broad agreement with earlier observations from western Kenya.^27^ In all age groups, parasite densities showed a modest decline over time, with considerable fluctuations in densities during individual infections. In Vietnam, 7% of ultra-low density *P falciparum* and *Plasmodium vivax* infections escalated into microscopy-positive infections.^6^ Longitudinal studies from Senegal and Ghana also showed marked fluctuations in the detectability of *P falciparum* infections over time.^2^ We observed similar oscillations in parasite densities. A unique component of our study was the longitudinal assessments of transmissibility to mosquitoes of these infections that fluctuated in density. We observed marked heterogeneity in transmission potential between and within individual infections, with four children (0·8% of our cohort) responsible for more than 60% of all infected mosquitoes. These four children and all individuals who were infectious to mosquitoes were microscopy-positive at least once during follow-up, suggesting that test-and-treat approaches could be used for controlling malaria transmission. However, 15·6% of the human infectious reservoir was attributable to infections that were submicroscopic at the time of feeding. This finding suggests that, unless repeated frequently, test-and-treat approaches will miss part of the human infectious reservoir.

Our study had several limitations. First, we used membrane feeding experiments that underestimate transmission potential compared with direct skin feeding.^28^ The lower sensitivity of membrane feeding was possibly compensated by the large number of mosquitoes we examined per feeding assay (median 69; IQR 62–81) that was much higher than natural mosquito exposure (average of two per indoor trapping night).^19^ Second, for logistical reasons we based our selection of participants for mosquito feeding on parasite carriage 4 weeks before feeds, and thus we did not measure infectiousness in the first weeks of incident asymptomatic infections. Third, health-seeking behaviour among our cohort participants, who had exceptionally good access to care, might not have reflected care in other settings. Some of the symptomatic malaria infections that we detected early after infection would possibly not have received such prompt treatment in other settings, and thus might have developed into chronic infections with transmissible gametocyte densities. Finally, we did mosquito feeding assays on a small number of symptomatic infections, affecting the precision of our estimates for this population.

In conclusion, our findings have several important implications for malaria control. We identified chronic asymptomatic infections with microscopically detectable parasitaemia as the most important drivers of transmission in our setting. These infections frequently occurred in school-aged children, supporting hypotheses that schoolchildren form an important and accessible reservoir that could be targeted by malaria control interventions, such as chemoprevention.^29^ Our finding of a small minority of asymptomatic individuals that are disproportionally important for transmission poses challenges for community-wide interventions. Identifying the populations that possibly sustain malaria transmission is key to progressing toward malaria elimination and to avoiding resurgence if regional malaria control deteriorates.

## Data sharing

Cohort data are available through a novel open-access clinical epidemiology database resource, ClinEpiDB. Data for the study done from Oct 4, 2017, to Oct 31, 2019 (referred to as PRISM2), can be found online.

## Declaration of interests

TB received a fellowship from the European Research Council (ERC-2014-StG 639776), and is further supported by a fellowship from the Netherlands Organisation for Scientific Research (Vidi fellowship NWO project number 016.158.306). MC is supported by the Fogarty International Center (P0529898 and TW009343) and the Centennial Travel Award from the American Society of Tropical Medicine and Hygiene. JIN is supported by the Fogarty International Center (TW010365). BG is a Chan Zuckerberg Biohub investigator. The open-access clinical epidemiology database resource (ClinEpiDB) platform is supported by the Bill & Melinda Gates Foundation (OPP1169785). All other authors declare no competing interests.
