# Supplementary material for: Sources of persistent malaria transmission in a setting with effective malaria control in eastern Uganda: a longitudinal, observational cohort study
Source: Lancet Infect Dis. 2021 Nov;21(11):1568–78. doi: 10.1016/S1473-3099(21)00072-4 (PMC8554388; doi:10.1016/S1473-3099(21)00072-4)
Supplement: Supplementary appendix [file mmc1.pdf]

THE LANCET
Infectious Diseases

Supplementary appendix

This appendix formed part of the original submission and has been peer reviewed. We post it as supplied by the authors.

Supplement to: Andolina C, Rek JC, Briggs J, et al. Sources of persistent malaria transmission in a setting with effective malaria control in eastern Uganda: a longitudinal, observational cohort study. *Lancet Infect Dis* 2021; published online June 16. https://doi.org/10.1016/S1473-3099(21)00072-4.

Supplementary appendix

This appendix formed part of the original submission and has been peer reviewed.
We post it as supplied by the authors.

Supplement to: Andolina C, Rek J, Briggs J et al. **Persistent malaria transmission from asymptomatic children and adults despite highly effective malaria control in eastern Uganda: an observational cohort study**

Appendix methods: Detailed statistical methods

Statistical analyses were performed in RStudio.[1,2] For data manipulation and figures, we used the dplyr[3] and ggplot2[4] packages, and for analysis requiring repeated measures we used the geepack package.[5] For analysis requiring random effects, we used the lme4 and lmerTest packages.[6,7]

Incidence estimation

Poisson regression with generalized estimating equations to account for repeated measures was used to estimate malaria incidence and incidence of asymptomatic infections. Any clones identified in the first 60 days of observation were considered to be baseline (persistent) infections. An incident asymptomatic infection was defined as any of the following after day 60 without associated symptoms: 1) any new clone (or group of clones) not seen in the patient prior, 2) a clone that had infected the patient before but had been cleared for 3 consecutive routine visits before re-infection, or 3) a positive qPCR result after three qPCR-negative visit.

Regression models for estimating associations

Multiple generalized linear models were used to model the association between parasitological and infectivity outcomes and covariates(s). Where appropriate, subject specific random intercepts were added to account for correlations between observations from the same individuals.

The general formula of the GLM used for the models was

$$g(y) = \beta' X + z_i + \epsilon_i \quad (1)$$

where y is the response and g is the link function as described, X is the vector of covariates, β is the vector of unknown regression coefficients (slopes) to be estimated, z_i is the random intercept for the ith individual and ϵ_i denotes the error term. The random intercepts are assumed to be gaussian distribution with a mean of zero and some constant variance as estimated in the model, i.e. $\epsilon_i \sim N(0, \sigma_z^2)$.

For models with a dichotomous or proportion response, we assumed a binomial distribution. Dichotomous responses were associated with a single Bernoulli trial. For proportion responses, we further specified the number of samples that contributed to this proportion being binomially distributed. All continuous densities (d) were log transformed as $\log_{10}(d + 0.001)$ to allow densities that could possibly be zero to be retained in the model. When zero densities were not needed to be included in the model (e.g. for analyses including clinical malaria cases only), the transformation was

$\log_{10}(d)$. A log link was used for proportion responses and a logit link used for dichotomous outcomes. For models with continuous responses, we assumed a gaussian distribution.

In particular, in our study, we used the mixed effects linear regression model to:

1. Model the effects of an interaction of fixed effects age group and year, and individual specific random intercepts, on the log transformed total parasite density.
2. Model the effect of log transformed parasite density as a fixed effect and individual specific random intercepts on the log transformed gametocyte density.
3. Model the fixed effect of age groups and individual specific random intercepts on the log transformed gametocyte density.

Further, using a mixed effects logistic regression, we modelled the effect of symptomatic status (whether the malaria episode was symptomatic or asymptomatic), as a fixed effect, on gametocyte prevalence with individual specific random intercepts.

To model the effect of total gametocyte density on the proportion of infected mosquitos, we considered only *P. falciparum* positive visits. We used a generalized linear regression model, where the outcome was the proportion of infected mosquitos and was assumed to come from a binomial distribution with n=number of dissections, and a log-link function. Log transformed total gametocyte density ($\log_{10}(Tgam + 0.001)$) was used as the explanatory variable. We did not include random intercepts in this model. The model for the estimated proportion of infected mosquitos ($\hat{y}$) was thus found to be

$$\log \hat{y} = -5.88 + 2.11 \times \log_{10}(Tgam + 0.001) \qquad (2)$$

This is similar to the model used by Bradley *et al.* [8]

Contribution to the infectious reservoir by infection category

The contribution of symptomatic, asymptomatic microscopy-detected, and PCR-detected infections to the infectious reservoir was estimated as the proportion of the infected population in each category weighted by the relative infectivity to mosquitoes of each category, as shown in **Supplemental note 3** in. [9] We first calculated the proportion of infections in each infection category amongst those with infections, i.e. w_s, w_m, w_p which are the proportions of infections in the symptomatic, asymptomatic microscopy-detected, and PCR-detected infections category, and $\sum_i w_i = 1$. From the data with

mosquito feeding, we calculated the proportion of infected mosquitos in each infection category as $p_i = \frac{\sum_j x_{ij}}{\sum_j n_{ij}}$ where $i \in \{s, m, p\}$ for the symptomatic, asymptomatic microscopy-detected, and PCR-detected infection categories respectively, j represents the samples in each infection category, x is the number of infected mosquitos and n is the number of dissections. The contributions to the infectious reservoir for each infection category was then calculated as $w_i \times p_i$. The combined infectious reservoir of the population is then calculated as:

$$w_s p_s + w_m p_m + w_p p_p$$

And the relative contribution to the infectious reservoir of category i where $i \in \{s, m, p\}$ is given as

$$\frac{w_i p_i}{\sum_i w_i p_i}.$$

	w_i	p_i	$w_i \times p_i$	$\frac{w_i p_i}{\sum_i w_i p_i}$	%
Symptomatic infection	0.028	0.0025	0.0000700	0.006	0.6%
Microscopy-detected asymptomatic infection	0.179	0.0571	0.0102209	0.838	83.8%
PCR-detected asymptomatic infection	0.793	0.0024	0.0019032	0.156	15.6%
			$\sum_i w_i p_i$ =0.0121941		

$\boldsymbol{w_i}$ are the proportions of individuals in each infection category. $\boldsymbol{p_i}\left(=\frac{\sum_j x_{ij}}{\sum_j n_{ij}}\right)$ is the proportion of infected mosquitos in each infection category, $\mathbf{w_i} \times \mathbf{p_i}$ is the contribution to the infectious reservoir for each infection category and $\frac{w_i p_i}{\sum_i w_i p_i}$ is the re-weighted contributions to the infectious reservoir such that $\sum_i \frac{w_i p_i}{\sum_i w_i p_i} = \mathbf{1}$, where the respective percentages are given in the last column.

Contribution to the infectious reservoir by age category

The contribution to the infectious reservoir in each age category (<5 years, 5-15 years and ≥16 years) was calculated as the proportion of infected mosquitos in each category weighted by the population proportion of each category. When mosquito feeding data was available, the proportion of infected mosquitos was $p_{ij} = \frac{x_{ij}}{n_{ij}}$ where j represents the different feeding samples, i represents each age category, x is the number of infected mosquitos and n is the number of dissected mosquitos. For *P.*

falciparum positive visits without feeding assays performed, the proportion infected mosquitos was imputed using the total gametocyte density and the relationship between total gametocyte density and proportion infected mosquitos from those with feeding assays, i.e. $p_{ij} = \exp(-5.88 + 2.11 \times \log_{10}(Tgam + 0.001))$ (equation (2) as given in figure 2B of the main manuscript). For all *P. falciparum* negative visits, the proportion infected mosquitos was specified as $p_{ij} = 0$. We then estimated the average proportion infected mosquitos within each age category, i.e. $p_i = \frac{\sum_j p_{ij}}{n_i}$ where $i \in \{1,2,3\}$ represents the age categories $< 5\ years, 5 - 15\ years,\ and \geq 16\ years$. The proportion of the population in each age category, i.e. w_1, w_2, w_3, was based on UN population census data for Uganda. [10]

	<5	5-15 years old	>16
2017	17,9%	31,7%	50,4%
2018	17,6%	31,6%	50,8%
2019	17,4%	31,4%	51,2%
mean	17,6%	31,6%	50,8%

The contributions to the infectious reservoir for each infection category was then calculated as $w_i \times p_i$. The combined infectious reservoir of the population is then calculated as:

$$w_1p_1 + w_2p_2 + w_3p_3$$

And the relative contribution to the infectious reservoir of age category i where $i \in 9$ is given as

$$\frac{w_ip_i}{\sum_i w_ip_i}.$$

	w_i	p_i	$w_i \times p_i$	$\frac{w_ip_i}{\sum_i w_ip_i}$	%
<5 years	0.176	0.0011	0.0002003931	0.258	25.8%
5-15 years	0.316	0.0014	0.0004559548	0.587	58.7%
16+ years	0.508	0.0002	0.0001209823	0.156	15.6%
			$\sum_i w_ip_i$ =0.0007773301		

$\boldsymbol{w_i}$ are the proportions of malaria cases by each age category, estimated from the three year average of the UN population census data shown in the table above. $\boldsymbol{p_i} = \frac{\sum_j p_{ij}}{n_i}$ is the average proportion of infected mosquitos in each age category where $\boldsymbol{p_{ij}}$ was calculated for each *P. falciparum* positive visit $\boldsymbol{j}$ where feeding was performed $\left(= \frac{x_{ij}}{n_{ij}}\right)$, estimated for each *P.*

falciparum positive visit $\boldsymbol{j}$ where feeding was not done using the predicted relationship between gametocyte density and proportion infected, and set to zero ($\boldsymbol{p_{ij}} = \boldsymbol{0}$) for each *P. falciparum* negative visit. $\boldsymbol{w_i} \times \boldsymbol{p_i}$ is the contribution to the infectious reservoir for each age category and $\frac{w_i p_i}{\sum_i w_i p_i}$ is the re-weighted contributions to the infectious reservoir such that $\sum_i \frac{w_i p_i}{\sum_i w_i p_i} = \boldsymbol{1}$, where the respective percentages are given in the last column.

Appendix methods: Determining copy numbers per gametocyte for CCp4 and PfMGET

In order to determine the copy numbers per gametocytes for CCp4 and PfMGET, purified male and female gametocytes standards were generated and purified by FACS from PfDynGFP/PfP45mCherry reporter line parasites. Gametocytes numbers were quantified by microscopy using a Bürker-Türk counting chamber and verified by 18s qPCR. Synthetic RNA standards were ran against purified male/female gametocytes to determine copies per gametocyte for each marker (CCCp4 and PfMGET). Two different volumes of purified gametocytes were used for extraction and each sample was analyzed 3 times in duplicate in 3 independent PCR runs. Standards from 3 runs were combined and used to make an overall standard curve for each sample volume. Linear regression was performed on log transformed data to assess the relationship between the two measures (gametocytes/ml and copies/ml) and the y intercept indicated the number of copies per gametocyte(conversion factor). The conversion factor from copies to gametocytes/ml was 14 for PfMGET and 19 per CCp4. [10]

As low levels of CCp4 (10,000 rings equal to 1 female gametocyte) and PfMEGET (100,000 rings equal to 1 male gametocytes) are present in rings, background ring noise was calculated by dividing the absolute number of asexual parasites by 10.000 for female gametocytes and by 100.000 for male gametocytes. The background was subtracted from the actual number of female and male gametocytes detected by multiplex qRT-PCR. Samples with an estimated density <0.01 gametocytes per µl were considered negative.

Table S1 Primer sequences and qPCR conditions for *var*ATS assay

***var*ATS Primer/Probe Sequences**

**Primers**	**Sequence**
Primer-fw (5’-3’)	**CCCATACACAACCAATTGGA**
Primer-rev (5’-3’)	**TTCGCACATATCTCTATGTCTATCT**
Probe (5’-3’)	**6-FAM- TTTTCCATAAATGGT-NFQ-MGB**

Table S2 Primer sequences and qPCR conditions for PfMGET CCp4 assay

PfMGET Primer/Probe Sequences

Primers	Sequence
Primer-fw (5’-3’)	**CGGTCCAAATATAAAAATCCTG**
Primer-rev (5’-3’)	**TGTG TAACG TATG ATTCATTTTC**
Probe (5’-3’)	**FAM-CAGCTCCAG CATTAAAACAC-BHQ1**

CCp4 Primer/Probe Sequences

Primers	Sequence
Primer-fw (5’-3’)	**CACATGAATATGAGAATAAAATTG**
Primer-rev (5’-3’)	**TAGGCGAACATGTGGAAAG**
Probe (5’-3’)	**TexasRed-AGCAACAACGGTATGTGCCTTAAAACG-BHQ2**

Appendix methods: Bioinformatics workflow.

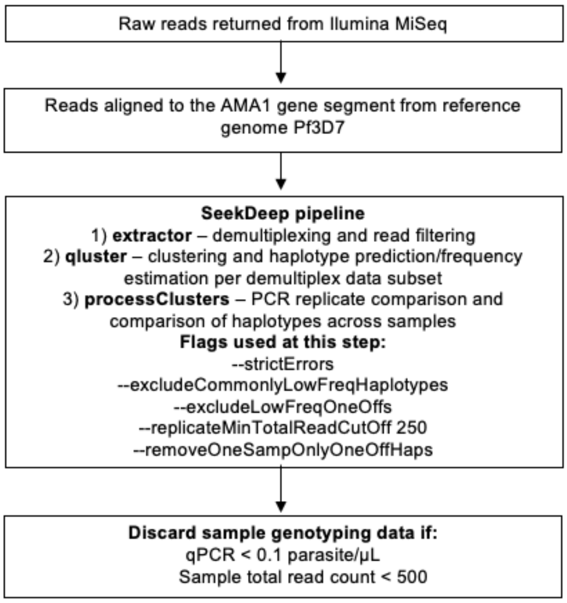

Sequencing library preparation and bioinformatics methods

Hemi-nested PCR was used to amplify a 236 base-pair segment of apical membrane antigen 1 (AMA-1) using a published protocol.[11] Samples were amplified in duplicate, indexed, pooled, and purified by bead cleaning. Sequencing was performed on an Illumina MiSeq platform (250bp paired-end). Data extraction, processing, and haplotype clustering were performed using SeekDeep,[12] followed by additional filtering as described elsewhere. [11]

Appendix Figure S1. Parasite density among the entire study cohort and the population selected for mosquito membrane feeding.

A violin plot showing that the distribution of parasite densities among participants selected for mosquito feeding assays (red) were similar to the distribution of parasite densities among the entire cohort (green).

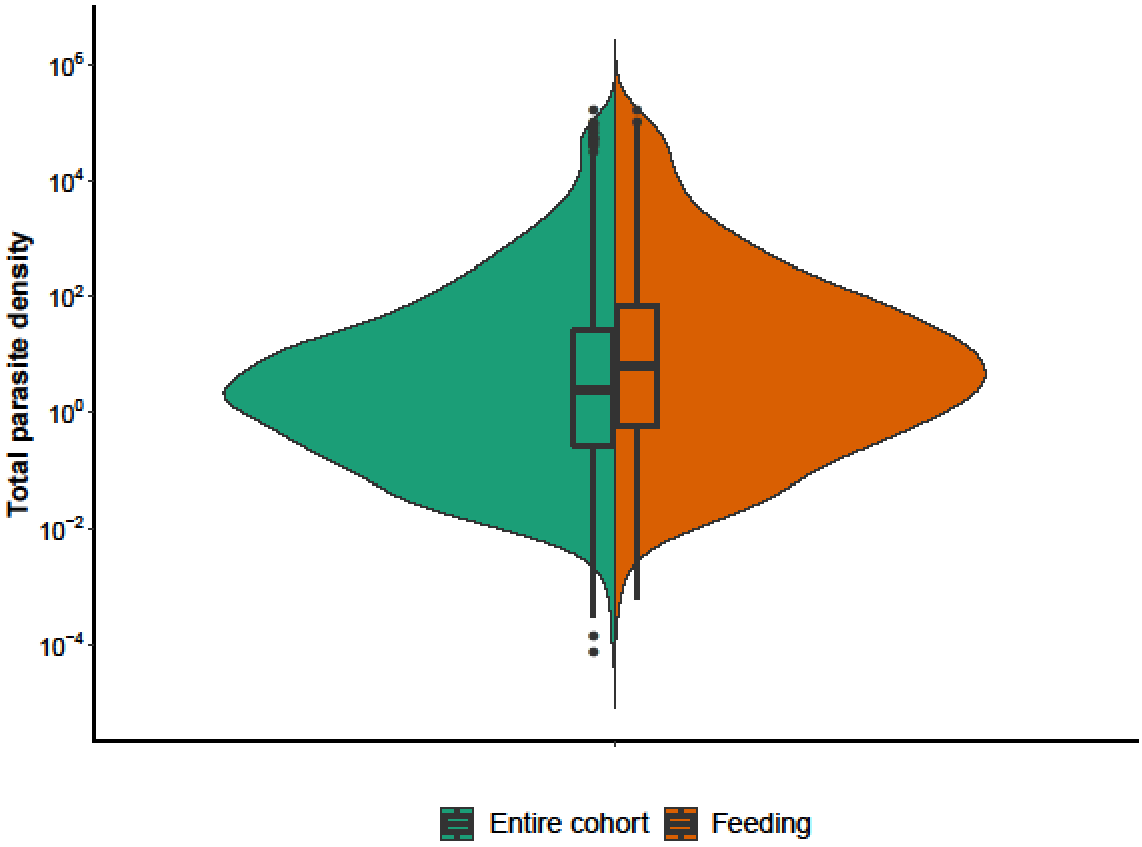

Appendix Figure S2. Parasite and gametocytes prevalence and density in relation to age.

Parasite prevalence (A) and density (B) by qPCR are highest in children aged 5-15 years. Gametocyte prevalence (C) is lowest in children <5 years whilst among gametocyte positive individuals gametocyte density (D) is highest in this age group. Individuals aged ≥16 years often have gametocyte densities below the minimum density to allow mosquito infections. The black line with grey shaded area is the same as Figure 2B in the main file.

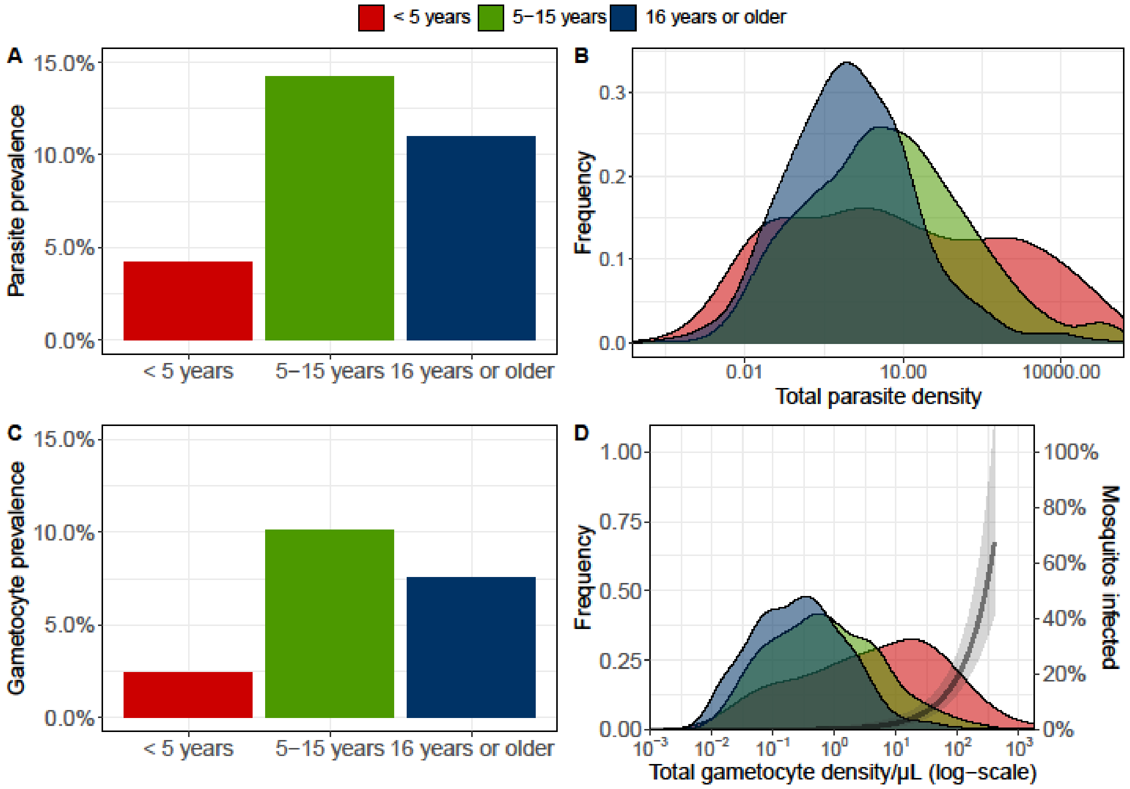

Appendix Figure S3. Gametocyte density and parasite clones recovered from blood and mosquito midguts in all infectious individuals.

Male and female gametocyte densities and the number of clones detected in blood and infected mosquitos are shown. Bars indicate infectious feeds, with each color representing the proportion of each unique *P. falciparum* clone. Total parasite density and clonal composition of in blood samples are presented in the bottom panel with each color indicating the contribution of a unique *P. falciparum* clone.

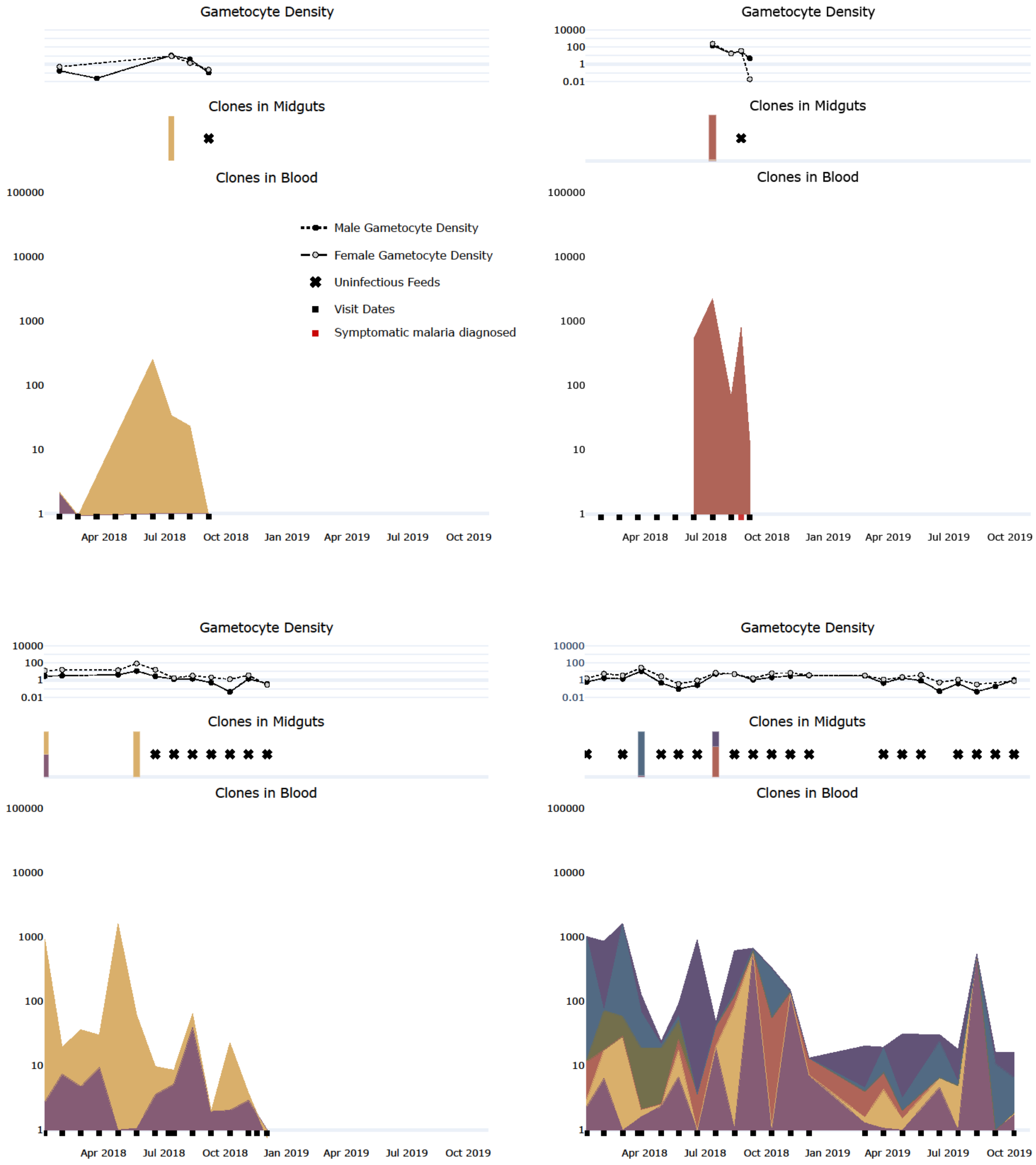
Gametocyte Density
Clones in Midguts
Clones in Blood
Male Gametocyte Density
Female Gametocyte Density
Uninfectious Feeds
Visit Dates
Symptomatic malaria diagnosed
10000
100
1
0.01
100000
10000
1000
100
10
1
Apr 2018
Jul 2018
Oct 2018
Jan 2019
Apr 2019
Jul 2019
Oct 2019

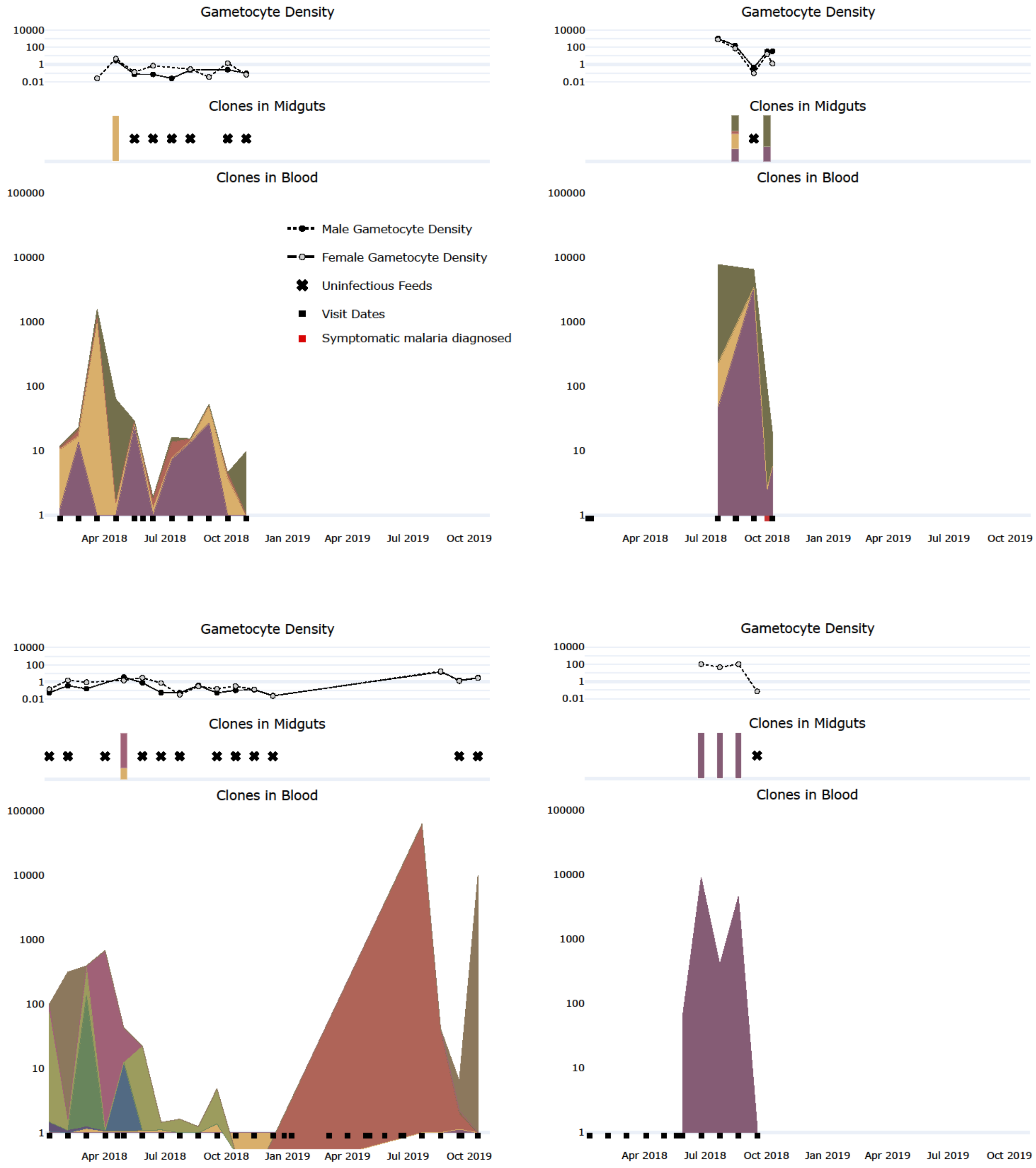
Gametocyte Density
10000
100
1
0.01
Clones in Midguts
Clones in Blood
100000
10000
1000
100
10
1
Male Gametocyte Density
Female Gametocyte Density
Uninfectious Feeds
Visit Dates
Symptomatic malaria diagnosed
Apr 2018
Jul 2018
Oct 2018
Jan 2019
Apr 2019
Jul 2019
Oct 2019

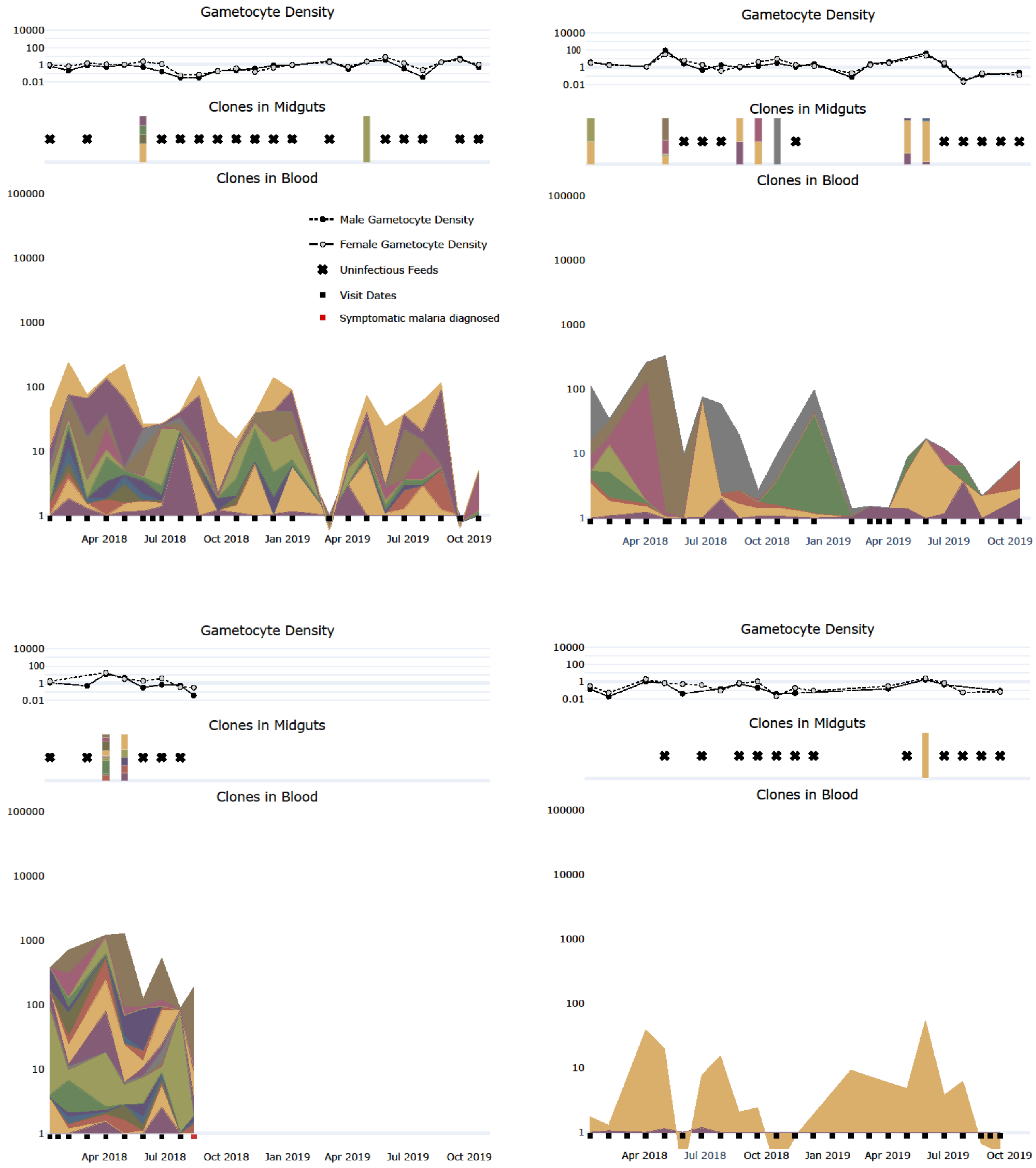
Gametocyte Density
10000
100
1
0.01
Clones in Midguts
Clones in Blood
100000
10000
1000
100
10
1
Male Gametocyte Density
Female Gametocyte Density
Uninfectious Feeds
Visit Dates
Symptomatic malaria diagnosed
Apr 2018
Jul 2018
Oct 2018
Jan 2019
Apr 2019
Jul 2019
Oct 2019

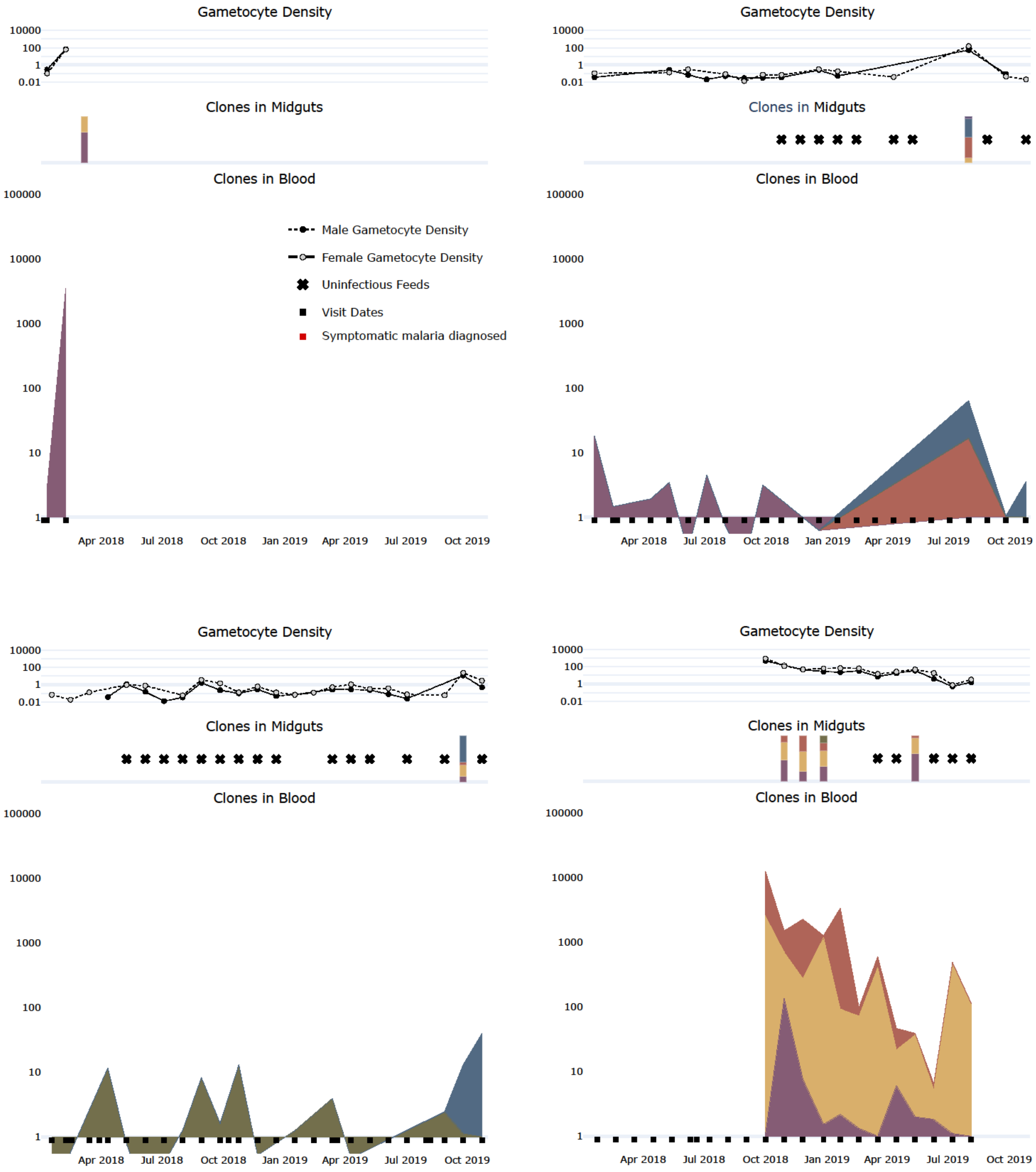
Gametocyte Density
10000
100
1
0.01
Clones in Midguts
Clones in Blood
100000
10000
1000
100
10
1
Male Gametocyte Density
Female Gametocyte Density
Uninfectious Feeds
Visit Dates
Symptomatic malaria diagnosed
Apr 2018
Jul 2018
Oct 2018
Jan 2019
Apr 2019
Jul 2019
Oct 2019

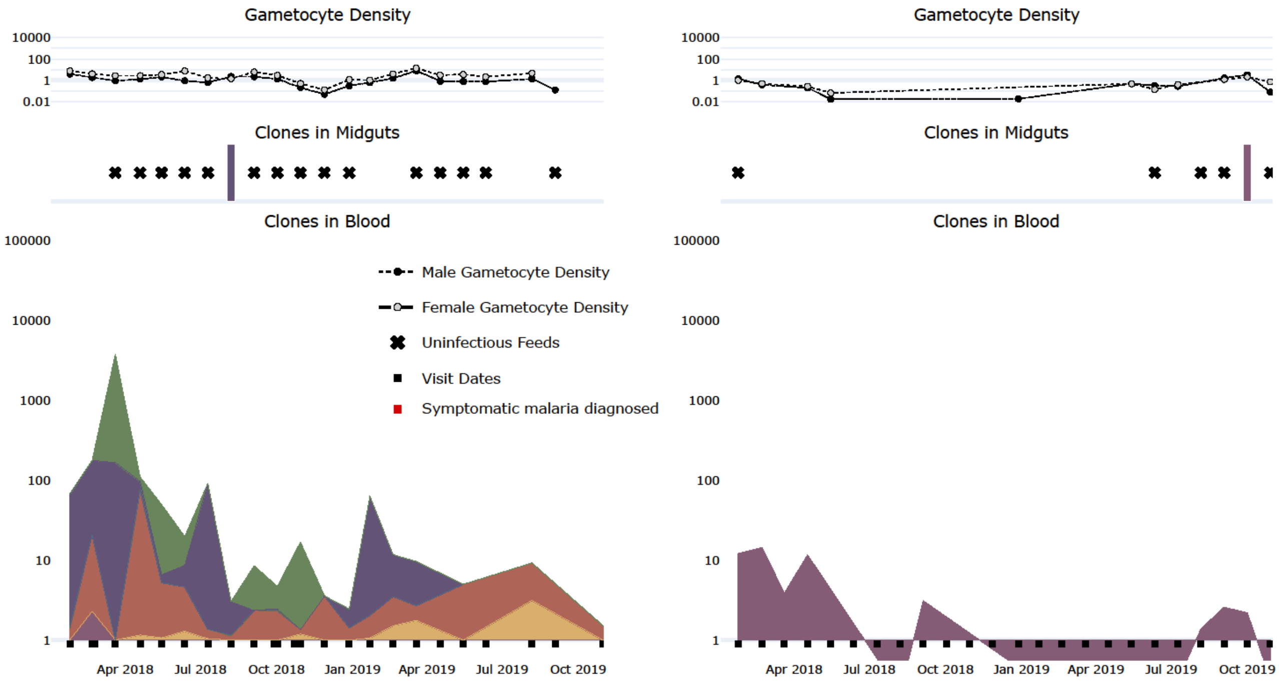
Gametocyte Density
10000
100
1
0.01
Clones in Midguts
Clones in Blood
100000
10000
1000
100
10
1
Male Gametocyte Density
Female Gametocyte Density
Uninfectious Feeds
Visit Dates
Symptomatic malaria diagnosed
Apr 2018
Jul 2018
Oct 2018
Jan 2019
Apr 2019
Jul 2019
Oct 2019
Gametocyte Density
10000
100
1
0.01
Clones in Midguts
Clones in Blood
100000
10000
1000
100
10
1
Apr 2018
Jul 2018
Oct 2018
Jan 2019
Apr 2019
Jul 2019
Oct 2019

Appendix Figure S4. Abundance of a clone in peripheral blood versus the abundance among all clones recovered in infected mosquitos. The blue line represents the regression of y ~ x with 95% confidence interval shaded in grey. Colours of dots indicate the clonal complexity in the blood sample.

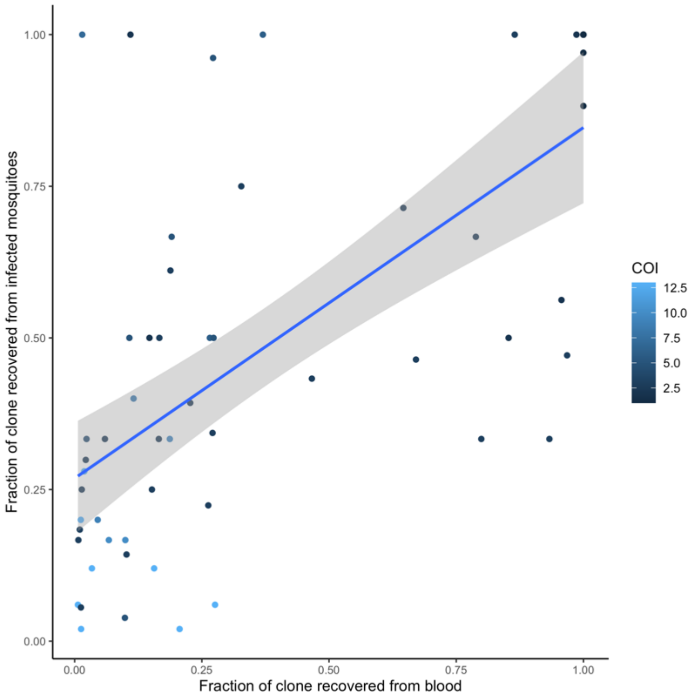

Data Accessibility.

Data from both cohort studies are available through a novel open-access clinical epidemiology database resource, ClinEpiDB. [13] Data for the study conducted from October 2011 through September 2017 (referred to as "PRISM1") can be found at https://clinepidb.org/ce/app/record/dataset/DS_0ad509829e . Data for the study conducted from October 2017 through October 2019 (referred to as "PRISM2") can be found at https://clinepidb.org/ce/app/record/dataset/DS_51b40fe2e2.

References:

1. Team RC. R: A language and environment for statistical computing. 2019.

https://www.R-project.org/.

2. Team R. RStudio: Integrated Development for R. RStudio, Inc., Boston, MA URL 2016. http://www.rstudio.com/

3. Hadley Wickham RF, Lionel Henry and Kirill Müller dplyr: A Grammar of

Data Manipulation. R package version 0.8.4. 2020. https://CRAN.R-project.org/package=dplyr.

4. Wickham H. ggplot2: Elegant Graphics for Data Analysis. New York: Springer International Publishing; 2016.

5. Højsgaard S HU, Yan J The R Package geepack for Generalized Estimating Equations. *Journal of Statistical Software* 2005; **15**(1): 1–11.

6. Douglas Bates MM, Ben Bolker, Steve Walker Fitting Linear Mixed-Effects Models Using lme4. *Journal of Statistical Software* 2015; **67**(1): 1-48

7. Kuznetsova A BP, Christensen RHB lmerTest Package: Tests in Linear Mixed Effects Models. *Journal of Statistical Software* 2017; **82**(13): 1-26.

8. Bradley J, Stone W, Da DF, et al. Predicting the likelihood and intensity of mosquito infection from sex specific Plasmodium falciparum gametocyte density. *Elife* 2018; **7**.

9. Adjalley SH, Johnston GL, Li T, et al. Quantitative assessment of Plasmodium falciparum sexual development reveals potent transmission-blocking activity by methylene blue. *Proc Natl Acad Sci U S A* 2011; **108**(47): E1214-23.

10. Wang CYT, Ballard E, Llewellyn S, et al. Assays for quantification of male and female gametocytes in human blood by qRT-PCR in the absence of pure sex-specific gametocyte standards. *Malar J* 2020; **19**(1): 218.

11. Briggs J, Teyssier N, Nankabirwa JI, et al. Sex-based differences in clearance of chronic Plasmodium falciparum infection. *Elife* 2020; **9**.

12. Hathaway NJ, Parobek CM, Juliano JJ, Bailey JA. SeekDeep: single-base resolution de novo clustering for amplicon deep sequencing. *Nucleic Acids Res* 2018; **46**(4): e21.

13. Ruhamyankaka E, Brunk BP, Dorsey G, et al. ClinEpiDB: an open-access clinical epidemiology database resource encouraging online exploration of complex studies. *Gates Open Res* 2019; **3**: 1661.
